# Suppression of lipopolysaccharide-induced COX-2 expression via p38MAPK, JNK, and C/EBPβ phosphorylation inhibition by furomagydarin A, a benzofuran glycoside from *Magydaris pastinacea*

**DOI:** 10.1080/14756366.2023.2287420

**Published:** 2023-12-07

**Authors:** Shiu-Wen Huang, Ming Jen Hsu, Hsiu-Chen Chen, Rita Meleddu, Simona Distinto, Elias Maccioni, Filippo Cottiglia

**Affiliations:** a Department of Pharmacology, School of Medicine, College of Medicine, Taipei Medical University, Taipei, Taiwan; b Department of Medical Research, Taipei Medical University Hospital, Taipei, Taiwan; c Graduate Institute of Medical Sciences, College of Medicine, Taipei Medical University, Taipei, Taiwan; d Department of Life and Environmental Sciences, University of Cagliari, Cittadella Universitaria di Monserrato, Monserrato, Italy

**Keywords:** COX-2, benzofurans, *Magydaris pastinacea*, p38MAPK, JNK, C/EBPβ

## Abstract

The phytochemical investigation of the methanol extract of the seeds of *Magydaris pastinacea* afforded two undescribed benzofuran glycosides, furomagydarins A-B (**1**, **2**), together with three known coumarins. The structures of the new isolates were elucidated after extensive 1D and 2D NMR experiments as well as HR MS. Compound **1** was able to inhibit the COX-2 expression in RAW264.7 macrophages exposed to lipopolysaccharide, a pro-inflammatory stimulus. RT-qPCR and luciferase reporter assays suggested that compound **1** reduces COX-2 expression at the transcriptional level. Further studies highlighted the capability of compound **1** to suppress the LPS-induced p38MAPK, JNK, and C/EBPβ phosphorylation, leading to COX-2 down-regulation in RAW264.7 macrophages.

## Introduction

Macrophages represent the primary innate immune cells responsible for defending the host from invading pathogens. Nonetheless, over-activated macrophages may heighten inflammation and tissue damage by producing abundant amounts of pro-inflammatory mediators. This dysregulation contributes to the progression of a variety of inflammatory diseases, including rheumatoid arthritis, atherosclerosis, inflammatory bowel disease, and sepsis^1^. Sepsis and its subsequent events remain the primary cause of mortality among critically ill patients^2^. Lipopolysaccharide (LPS), the major component of the outer membrane of Gram-negative bacteria, emerges as a prevailing catalyst for sepsis progression^3^. LPS can lead to macrophage activation via toll-like receptor 4 (TLR4) signalling followed by the activation of mitogen-activated protein kinase (MAPK) signalling cascades^4^^,^^5^. MAPKs, particularly p38MAPK and c-Jun N-terminal kinase (JNK), can contribute to the pathogenesis of immune and inflammatory disorders via increasing expression of pro-inflammatory mediators in cells in response to inflammatory stimuli^6^. There is increasing evidence that activation of p38MAPK or JNK signalling causes transcription factor CCAAT/enhancer-binding protein (C/EBP) activation, which in turn transactivates a number of pro-inflammatory mediators, including cyclooxygenase-2 (COX-2)^7–10^. Cyclooxygenases (COX) are a family of isozymes responsible for catalysing the conversion of arachidonic acid to prostanoids. However, contrary to cyclooxygenase-1 (COX-1), which is constitutively expressed in most normal tissues and involved in physiological processes, cyclooxygenase-2 (COX-2), scarcely present in most normal cells, can be highly induced by inflammation and cancer^11^. Prostaglandins are crucial in maintaining homeostasis within the body and mediating inflammatory responses^12^. As a crucial inducible enzyme for catalysing the biosynthesis of prostaglandins, COX-2 was recognised as a promising target for treating inflammation ^13^. Therefore, attenuation of aberrant macrophage activation and COX-2 expression represents a rational strategy in the treatment of inflammatory disorders.

**Figure 1.**
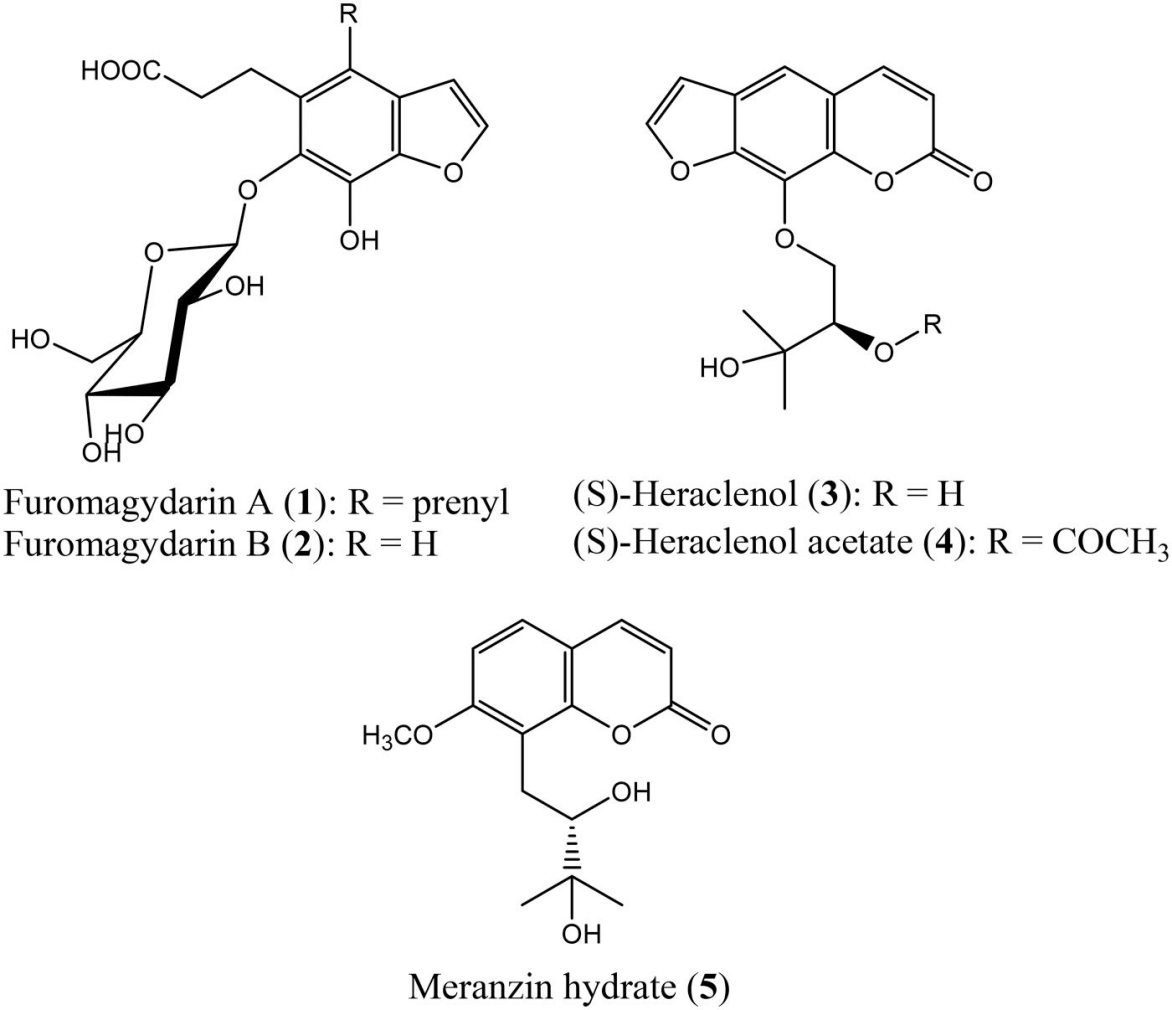
Chemical structures of the isolated compounds.

*Magydaris pastinacea* (Lam.) Paol. (Apiaceae) is a species that grows in few regions of the Mediterranean area, especially in Sardinia, Sicily, and Corse. *M. pastinacea* is a rich source of coumarins and furocoumarins endowed with cytotoxic, antibacterial, and anticoagulant activities^14^^,^^15^. In recent work, we reported the phytochemical investigation of the petroleum ether and ethyl acetate extracts of *M. pastinacea* seeds affording a series of coumarins and furocoumarins^16^. The isolated compounds showed a selective inhibitory activity towards the human carbonic anhydrases IX and XII which are well-known tumor-associated isoforms and are overexpressed in many hypoxic tumours showing a restricted expression in normal tissues^17^. In this investigation, the purification of the methanol extract of *M. pastinacea* seeds by vacuum-liquid chromatography (VLC), column chromatography (silica gel and Sephadex LH 20) and semi-preparative HPLC yielded two undescribed benzofuran glycosides that were trivially named furomagydarins A (**1**) and B (**2**) along three known coumarins, (S)-heraclenol (**3**), (S)-heraclenol acetate (**4**) and (S)-meranzin hydrate (**5**) (Figure 1). Given the interesting anti-inflammatory activity showed by natural as well as synthetic benzofurans^18^ through reducing mRNA COX-2 expression^19–21^, we decided to evaluate the new compounds capability to inhibit the COX-2 expression in RAW264.7 macrophages exposed to LPS.

## Materials and methods

### General experimental procedures

UV spectra were recorded on a GBC Cintra 5 spectrophotometer. NMR spectra of all isolated compounds were recorded at 25 °C on Bruker Avance III HD 600 high-resolution spectrometer operating at 600 MHz for ^1^H and 100 MHz for ^13^C. Spectra were measured in CD_4_O and CDCl_3_ and referenced against residual non-deuterated solvents. HRESIMS were measured on an Agilent 6520 Time of Flight (TOF) MS instrument. Column chromatography was carried out under TLC monitoring using silica gel (40–63 µm, Merck), and Sephadex LH-20 (25–100 µm, Pharmacia). For vacuum-liquid chromatography (VLC), silica gel (40–63 µm) (Merck) or silica gel RP-18 (Sigma-Aldrich) was used. TLC was performed on silica gel 60 F254 or RP-18 F254 (Merck). LiChrolut RP-18 (40–63 µm) 500 mg, 3 ml (Merck) Solid Phase Extraction (SPE) cartridges were also used. Semi-preparative HPLC was conducted by means of a Varian 920 LH instrument fitted with an autosampler module with a 1000 µL loop. The peak purities were monitored using a dual-wavelength UV detector settled at 254 and 366 nm or a Varian 356-LC refractive index detector. The columns were a 250 × 10 mm Kinetex C-18 core-shell, particle size 5 µm (Phenomenex) and a 250 × 4.6 mm Chiralpak IA, particle size 5 µm (Daicel).

### Plant material, extraction, and isolation

The seeds of *M. pastinacea* were collected in July 2017 at Siniscola (Nuoro), Sardinia, Italy. The plant material was identified by Prof. Marco Leonti (University of Cagliari, Department of Biomedical Sciences). A voucher specimen (No. 0485) was deposited in the Herbarium of the Department of Life and Environmental Science, Drug Sciences Section, University of Cagliari.

Air-dried and powdered seeds of *M. pastinacea* (720 g) were ground and extracted with petroleum ether (3.5 L) by percolation at room temperature to give 77.6 g dried extract. The remaining plant material was then extracted with EtOAc (3 L) and then with MeOH (2 L) giving 42 g and 49.8 g, respectively.

The MeOH extract was subjected to VLC (silica gel, 120 g, 40–63 µm) using a step gradient of DCM/MeOH/H_2_O (10: 0: 0 to 0: 9: 1, 500 ml each) to yield 28 fractions. Based on the TLC similarities, identical fractions were combined to give a total of eight fractions (F1-F8). F5 (5 g) was subjected to VLC (RP-18, 10 g) using a step gradient of H_2_O/ACN (7: 3 to 0: 10, 50 ml each) to give 15 fraction that, after TLC, were combined in five fractions (F5.1–F5.5). F5.3 (80 mg) was purified by RP-18 HPLC using H_2_O/ACN/trifluoroacetic acid (TFA) as eluents (7.5: 2.5: 0.1, flow 2.8 ml/min) to furnish compound **2** (5.6 mg, t_R_ 20.1 min). F5.4 (70 mg) was subjected to RP-18 HPLC using H_2_O/ACN/TFA as eluents (6.5: 3.5: 0.1, flow 2.8 ml/min) to give compound **1** (6.5 mg, t_R_ 11 min). F3 (1.3 g) was chromatographed by column chromatography (CC) over silica gel using DCM/EtOAc (4: 6) as eluent, to furnish compound **3** (77.2 mg), **4** (9.1 mg) and **5** (26.3 mg).

Furomagydarin A (**1**): white amorphous solid; UV (CH_3_OH) λmax (log ε): 249 nm; ^1^H (CD_4_O, 600 MHz) and ^13^C (CD_4_O, 100 MHz) NMR, see Table 1; HRTOFESIMS *m/z* 451.1613 [M - H]^−^ (negative mode) (calcd 451.1610).

**Table 1.** ^1^H (600 MHz) and ^13^C (100 MHz) NMR data for compounds **1**–**2** (CD_4_O, δ in ppm).

Compound **1**			Compound **2**	
Position	δ_C_, type	δ_H_ (*J* in Hz)	δ_C_, type	δ_H_ (*J* in Hz)
2 3	146.2, CH 106.6, CH	7.69, d (2.4) 6.77, d (2.4)	145.5, CH 106.2, CH	7.69, d (2.4) 6.72, d (2.4)
4	123.5, C		110.0, CH	6.94, s
5	129.1, C		130.3, C	
6	142.6, C		140.7, C	
7	135.2, C		135.3, C	
8	144.0, C		143.1, C	
9	127.1, C		125.8, C	
1'a	23.6, CH_2_	3.11, m	25.9, CH_2_	3.11, m
1'b		3.27, m		3.24, m
2′	35.9, CH_2_	2.58, m	35.0, CH_2_	2.68, m
3′	177.9, C		176.5, C	
1′'	29.7, CH_2_	3.53, d (6.6)		
2′'	124.9, CH	5.11, t (6.6)		
3′'	132.2, C			
4′'	18.1, CH_3_	1.83, s		
5′'	25.8, CH_3_	1.71, s		
1′''	108.0, CH	4.69, d (7.8)	106.8, CH	4.68, d (7.8)
2′''	75.7, CH	3.57, t (8.4)	74.3, CH	3.57, t (8.4)
3′''	78.1, CH	3.47, m	76.6, CH	3.47, m
4′''	71.1, CH	3.45, m	69.7, CH	3.45, m
5′''	78.6, CH	3.34, m	77.3, CH	3-34, m
6′''a	62.5, CH_2_	3.76, dd (5.4, 12)	61.1, CH_2_	3.76, dd (5.4, 12)
6′''b		3.90, dd (2.4, 12)		3.90, dd (2.4, 12)

Furomagydarin B (**2**): white amorphous solid; UV (CH_3_OH) λmax (log ε): 242 nm; ^1^H (CD_4_O, 600 MHz) and ^13^C (CD_4_O, 100 MHz) NMR, see Table 1; HRTOFESIMS *m/z* 383.0983 [M - H]^−^ (negative mode) (calcd 383.0984).

#### Acid hydrolysis of compounds 1 − 2 and determination of sugar configuration

Each compound (1.5 mg) was treated with 1 N HCl (5 ml) (80 °C, 2 h). After extraction with EtOAc (3 × 20 ml), the aqueous layer was neutralised with 0.5 M NaOH and freeze-dried. The water fraction was evaporated, dissolved in MeOH and analysed by chiral-phase HPLC equipped with a CHIRALPAK IA column (250 × 4.6 mm) and a refractive index detector, using an isocratic of n-hexane: EtOH: TFA (800: 200: 0.25) (0.5 ml/min). For both compounds (**1** and **2**), the sugar was identified as D-glucose by comparing its retention time with that of authentic L-glucose and D-glucose.

### Reagents

Lipopolysaccharides purified by phenol extraction from Escherichia coli 0127:B8 was purchased from Sigma (St. Louis, MO). DMEM, opti-MEM, foetal bovine serum (FBS), penicillin, streptomycin, transfection reagent Turbofect™ and all cell culture reagents were purchased from Invitrogen (Carlsbad, CA, U.S.A.). Antibodies against p38MAPK, p38MAPK phosphorylated at Thr180/Tyr182, JNK1/2, JNK1/2 phosphorylated at Thr183/Tyr185, C/EBPβ, C/EBPβ phosphorylated at Thr 235, were purchased from Cell Signalling (St. Louis, MO, USA). Antibodies specific for α-tubulin and COX-2 were from Novus Biologicals (Centennial, CO, USA). Anti-mouse and anti-rabbit immunoglobulin G (IgG)-conjugated horseradish peroxidase (HRP) antibodies were purchased from GeneTex Inc (Irvine, CA). Murine COX-2 promoter with wild type construct (native −966/+23) cloned into pGL3-basic vector (Promega) were kindly provided by Dr. Byron Wingerd (Michigan State University, East Lansing, MI, USA). pGL3-COX-2–3’UTR reporter construct was kindly provided by Dr. Paloma Martin-Sanz (Instituto de Biomedicina de Valencia Spanish Council for Scientific Research (CSIC), Valencia, Spain). κB-Luc, Renilla-luc and Dual-Glo luciferase assay system were purchased from Promega (Madison, WI, U.S.A.). The enhanced chemiluminescence detection kit was from Millipore (Billerica, MA, U.S.A.). All materials for immunoblotting were purchased from Bio-Rad (Hercules, CA, U.S.A.). All other chemicals were obtained from Sigma (St. Louis, MO).

### Cell culture

The RAW 264.7 mouse macrophage cell line was purchased from the Bioresource Collection and Research Centre (Hsinchu, Taiwan), and cells were maintained in DMEM (Invitrogen, Carlsbad, CA, USA) containing 10% FBS, 100 U/mL of penicillin G, and 100 μg/mL streptomycin in a humidified 37 °C incubator.

### Immunoblotting

Immunoblotting was performed as described previously^9^. Briefly, cells were lysed in extraction buffer containing 0.5% NP-40, 2 mM PMSF, 5 mM DTT, 0.05 mM, 10 mM Tris (pH 7.0), 140 mM NaCl, pepstatin A, and 0.2 mM leupeptin. Equal amounts of protein samples were subjected to sodium dodecylsulfate polyacrylamide gel electrophoreses (SDS-PAGE) and transferred onto a nitrocellulose (NC) membrane (Pall Corporation, Washington, NY, U.S.A.). After blocking in a 5% non-fat milk-containing blocking buffer (150 mM NaCl, 20 mM Tris-HCl, and 0.02% Tween 20; pH 7.4) for 1 h, proteins were recognised using specific primary antibodies for 2 h, followed by the incubated with horseradish peroxidase-conjugated secondary antibodies for another 1 h. Immunoreactivity was detected based on enhanced chemiluminescence according to instructions by the manufacturer. Quantitative data were obtained using a computing densitometer with a scientific imaging system (Biospectrum AC System, UVP).

### Reverse-transcription-quantitative real-time PCR (RT-qPCR)

RT-qPCR analyses were performed as described previously^22^. Total RNA was isolated from cells using Trizol reagent (Invitrogen, Carlsbad, CA, U.S.A) and the GoScript™ Reverse Transcription System (Promega, Madison, WI, USA) was employed for cDNA synthesis according to the manufacturer’s instructions. The cDNAs were stored at −20 °C until qPCR was performed in the StepOne Real-Time PCR systems (Applied Biosystems, Grand Island, NY, U.S.A.). Real-time PCR was performed with the GoTaq qPCR Master Mix (Promega, Madison, WI, USA) and cycling condition was as follows: hot-start activation at 95 °C for 2 min, followed by 40 cycles of denaturation at 95 °C for 15 s, annealing/extension at 60 °C for 60 s respectively. Primers used for amplification of the COX-2 and GAPDH fragments were as follows: COX-2, sense 5′-CCCCCACAGTCAAAGACACT-3′ and antisense 5′-CTCATCACCCCACTCAGGAT-3′; and GAPDH, sense 5′-CCTTCA TTGACCTCAACTAC-3′ and antisense 5′-GGAAGGCCATGCCAGTGAGC-3′. GAPDH was used as the internal control.

### Cell viability assay (MTT assay)

RAW264.7 macrophages (10^5^ cells per well) were treated with vehicle or furomagydarin A (10 μM) for 24 h. Cell viability was measured by the colorimetric 3–(4,5-dimethylthiazol-2-yl)-2,5-diphenyl tetrazolium bromide (MTT) assay as described previously^22^.

### Cell viability assay (Trypan blue exclusion assay)

RAW264.7 macrophages (10^5^ cells per well) were treated with vehicle or furomagydarin A (10 μM) for 24 h. Trypan blue dye exclusion assay was performed as described previously^8^. Live cells excluded the dye whereas the dye entered and stained the dead cells blue in colour. Both stained and unstained cells were counted, and cell viability was calculated using the formula: cell viability (%) = 100×(live cells)/(dead cells + live cells).

### Transfection in RAW264.7 macrophages and dual luciferase reporter assay

RAW264.7 macrophages were transfected with COX-2-luc or COX-2–3’UTR-luc plus renilla-luc using Turbofect™ transfection reagent (Invitrogen, Carlsbad, CA, U.S.A.) according to the manufacturer’s instructions. After transfection, cells with and without treatments were then harvested. The luciferase activity was determined using a Dual-Glo luciferase assay system kit (Promega) according to the manufacturer’s instructions. Normalisation was performed with renilla-luciferase activity as the basis.

### Statistical analysis

Results are presented as the mean ± SE from at least five independent experiments. Western blot post-analysis was carried out by the band intensity quantification via Fiji^23^. The intensity of the target protein was then normalised by the internal control protein (GAPDH) or its relative total protein (phosphorylated form over total protein). For the better comparison of the ratio distribution of each replicated experiment, the final results were illustrated as the fold change to the control group (vehicle-treated group), where the control group was represented as 1 (100%) in the statistical graph. For the MTT assay, the viability of vehicle-treated cells (control group) was considered 100%, and the viability of cells in the presence of furomagydarin A was expressed as a percentage of the control. One-way analysis of variance (ANOVA) followed by, when appropriate, the Newman-Keuls test was used to determine the statistical significance of the difference between means. A *p* values of < 0.05 was considered statistically significant.

## Results and discussion

### Structure elucidation

**Figure 2.**
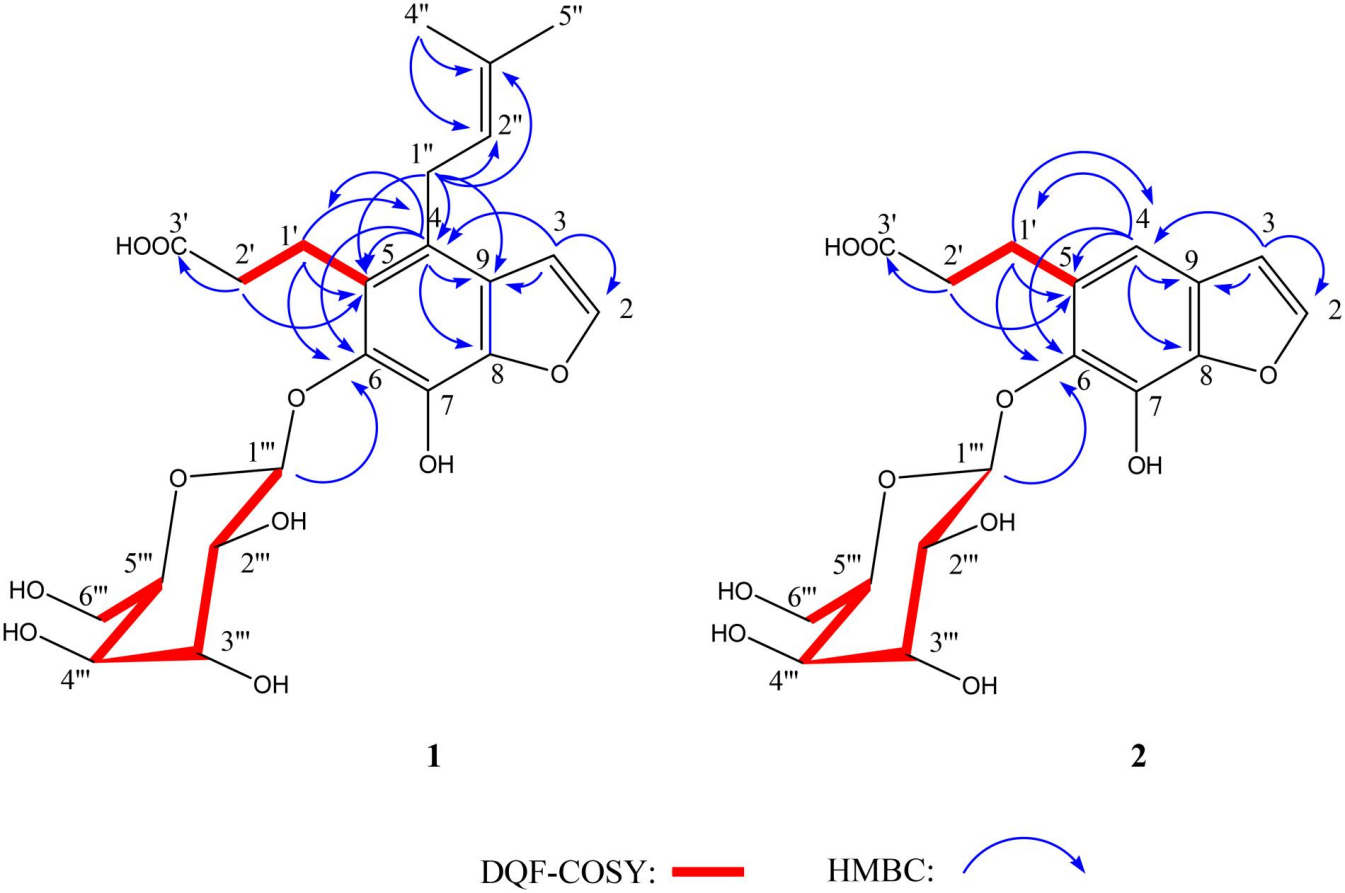
Main HMBC and DQF-COSY correlations of compounds **1**–**2**.

Compound **1** was obtained as a white, amorphous solid. The ^13^C NMR spectrum of compound **1** exhibited 22 carbon signals, which were sorted by multiplicity-edited HSQC experiment into two CH_3_, four CH_2_, eight CH, and eight quaternary carbons (Table 1). These data were in agreements with the molecular formula C_22_H_28_O_10_ and consistent with the measured pseudomolecular ion at *m/z* 451.1613 [M - H]^−^ (calcd 451.1610) in HRTOFESIMS (negative mode). The ^1^H NMR spectrum of compound **1** in CD_4_O showed two signals at 6.77 (1H, d, *J* = 2.4 Hz) and 7.69 (1H, d, *J* = 2.4 Hz) ppm, characteristic of a disubstituted furan ring belonging to a furocoumarin (Table 1). However, in the ^13^C NMR spectrum, the absence of a carbonyl group in the range of 160–161 ppm suggested the presence of a benzofuran skeleton instead of a furocoumarin. ^1^H − ^1^H COSY spectrum revealed correlations between the methylene protons at 2.58 (2H, m) and those at 3.11 (1H, m) and 3.27 (1H, m) ppm (Figure 2) that, besides the cross-peaks observed in the HMBC spectrum between the methylene protons at δ 2.58 and the carbons at 177.9 (C-10) and 23.6 (C-8) ppm and between the methylene at 3.11 and 3.27 ppm and the carbons at 35.9 (C-9), 123.5 (C-4), 129.1 (C-5) and 142.6 (C-6) ppm (Figure 2), led to the identification of an acid propionic chain linked to an aromatic nucleus. Furthermore, the ^1^H NMR spectrum showed two tertiary methyl groups at δ = 1.71 (3H, s) and 1.83 (3H, s), a methine at δ = 5.11 (1H, t, *J* = 6.6 Hz) and a methylene at δ = 3.53 (2H, d, *J* = 6.6 Hz) suggesting the presence of a prenyl group. In addition to these signals, the ^1^H NMR spectrum exhibited resonances for five methines at δ 3.33 (1H, m), 3.45 (1H, m), 3.47 (1H, t, *J* = 8.4 Hz), 3.57 (1H, d, *J* = 7.8 Hz), 4.69 (1H, d, *J* = 7.8 Hz) and one oxymethylene at δ 3.76 (1H, dd, *J* = 5.4, 12 Hz) and 3.90 (1H, dd, *J* = 2.4, 12 Hz) that are directly linked to the carbons at δ 78.6, 71.1, 78.1, 75.7, 108.0 and 62.5, respectively, as deduced by HSQC experiment. These data were attributable to a β-glucosyl moiety where the β-configuration was assigned on the basis of the anomeric carbon chemical shift (δ 108.0) and the large coupling constant of the anomeric proton (δ 4.69, *J* = 7.8 Hz). HMBC correlations from the methine proton at δ 7.69 to C-9 (δ 127.1), C-8 (δ 144.0) and from H-1′' (δ 3.53) to C-4 (δ 123.5), C-5 (δ 129.1), C-9 (δ 127.1) and C-2′' (124.9) fixed the prenyl chain at position 4 of the benzofuran, whereas cross peaks from the methylene protons at δ 3.11 (1H, m) and 3.27 (1H, m) to C-4 (δ 123.5), C-5 (δ 129.1) and C-6 (δ 142.6) indicated the placement of the propionic acid chain at position 4 (Figure 2). The glucosyl ring, through β-glycosidic linkage, was attached to the C-6, as judged from the HMBC correlation signal from the anomeric proton at δ 4.69 to C-6 (δ 142.6) (Figure 2). The D-glucopyranose moiety was identified by comparison with an authentic sample by chiral HPLC analysis after acid hydrolysis. The structure of **1** was therefore assigned as 4–(3-methylbut-2-en-1-yl)-5-carboxyethyl-6,7-dihydroxybenzofuran 6-O-β-D-glucopyranoside and trivially named furomagydarin A.

Compound **2** was obtained as a white, amorphous solid. The ^13^C NMR spectrum of compound **2** exhibited 17 carbon signals, which were sorted by multiplicity-edited HSQC experiment into three CH_2_, eight CH, and six quaternary carbons (Table 1). These data were in agreements with the molecular formula C_17_H_20_O_10_ and consistent with the measured pseudomolecular ion at *m/z* 383.0983 [M - H]^−^ (calcd 383.0984) in HRTOFESIMS (negative mode). The ^1^H NMR spectrum of compound **2** resembled to that of furomagydarin A, suggesting the presence of a propionic acid chain and a glucosyl ring attached to the benzofuran nucleus. However, in the 1H NMR spectrum of **2** an additional methine at δ 6.94 (1H, s) instead of the tertiary methyl groups at δ 1.71 and, 1.83, of the methine at δ 5.11 and of the methylene at δ 3.53 in furomagydarin A was observed in **2** (Table 1). HMBC correlations between the methine proton at δ 6.94 and C-1′ (δ 25.9), C-3 (δ 106.2), C-9 (δ 125.6), C-6 (δ 140.7) and C-8 (δ 143.0) confirmed the presence of an aromatic proton instead of a prenyl moiety (Figure 2). The D-glucopyranose moiety was identified by comparison with an authentic sample by chiral HPLC analysis after acid hydrolysis. The structure of 2 was therefore assigned as 5-carboxyethyl-6,7-dihydroxybenzofuran 6-O-β-D-glucopyranoside and trivially named furomagydarin B.

The spectroscopic (^1^H- and ^13^C-NMR and MS) and physical data (optical rotation) of the known compounds (S)-heraclenol (**3**)^24^, (S)-heraclenol acetate (**4**)^16^ and (S)-meranzin hydrate (5)^25^ were in agreement with the literature data.

### Biological activities

#### Furomagydarin A reduces LPS-induced COX-2 expression in RAW264.7 macrophages

**Figure 3.**
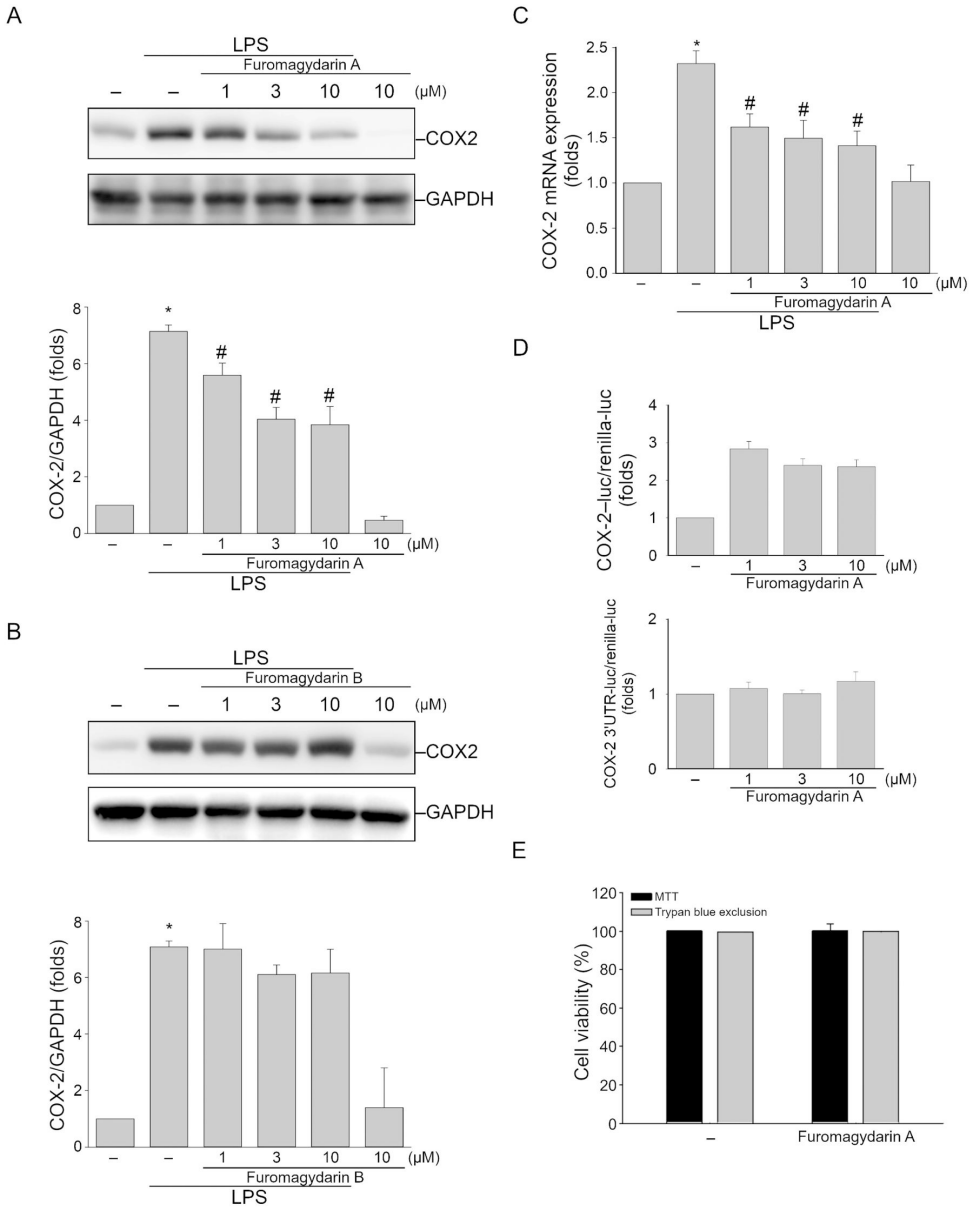
Furomagydarin A reduced COX-2 expression in LPS-stimulated RAW264.7 macrophages. (A) Cells were treated with vehicle or indicated concentrations of furomagydarin A for 30 min, followed by the treatment with LPS (100 ng/ml) for another 24 h. The COX-2 level was determined by immunoblotting. Each column represents the mean ± SEM of seven independent experiments. (B) Cells were treated with vehicle or indicated concentrations of furomagydarin B for 30 min, followed by the treatment with LPS (100 ng/ml) for another 24 h. The COX-2 level was determined by immunoblotting. Each column represents the mean ± SEM of six independent experiments. (C) Cells were treated with furomagydarin A (1–10 μM) for 30 min, followed by the treatment with LPS (100 ng/ml) for another 6 h. The extent of COX-2 mRNA was determined by an RT-qPCR assay as described in the ‘Materials and methods’ section. Each column represents the mean ± SEM of eight independent experiments. (D) Cells were transiently transfected with COX-2-luc or COX-2–3’UTR-luc and renilla-luc for 24 h. Luciferase activity was determined after treatment with LPS (100 ng/ml) for another 24 h. Data represent the mean ± SEM of eight independent experiments performed in duplicate. *P < 0.05, compared with the control group; #*P* < 0.05, compared with the group treated with LPS alone. (E) Cells were treated with furomagydarin A (10 μM) for 24 h. Cell viability was then determined using MTT and trypan blue exclusion assays. Data represent the mean ± SEM of four independent experiments performed in duplicate.

To examine the anti-inflammatory properties of furomagydarins A (**1**) and B (**2**), their effect on COX-2 levels was evaluated in RAW264.7 macrophages with 24-h LPS stimulation. The result indicated that furomagydarin A treatment resulted in remarkable decrease in COX-2 protein expression in LPS-stimulated RAW264.7 macrophages in a concentration-dependent manner (Figure 3A) with a IC_50_ value of 5.3 µM, while furomagydarin B could not significantly inhibit LPS-induced COX-2 expression (Figure 3B). RT-qPCR and luciferase reporter assays were employed to reveal inhibitory actions of furomagydarin A on COX-2 expression at the transcriptional level. As shown in Figure 3(C), COX-2 mRNA expression was highly increased after 6-h LPS stimulation, and it was inhibited by furomagydarin A treatment in a concentration-dependent manner. Luciferase reporter assay had further revealed that furomagydarin A possessed its inhibitory effect on LPS-induced COX-2 promoter luciferase activity (Figure 3D, upper panel) but no post-transcriptional 3’UTR cleavage of COX-2 mRNA was involved (Figure 3D, lower panel). MTT assay and trypan blue exclusion assay were used to examine whether furomagydarin A affects the cell viability of RAW264.7 macrophages. As shown in Figure 3(E), 24 h exposure to furomagydarin A at 10 μM was without effects on cell viability in RAW264.7 macrophages (Figure 1D). These results indicate that furomagydarin A reduces COX-2 expression, at least in part, by transcriptional regulation. It also suggests that furomagydarin A’s inhibitory actions on LPS-induced COX-2 expression is not attributed to its cytotoxic effects on RAW264.7 macrophages.

#### Furomagydarin A inhibits LPS-induced phosphorylation of p38MAPK, JNK1/2 and C/EBPβ in RAW264.7 macrophages

**Figure 4.**
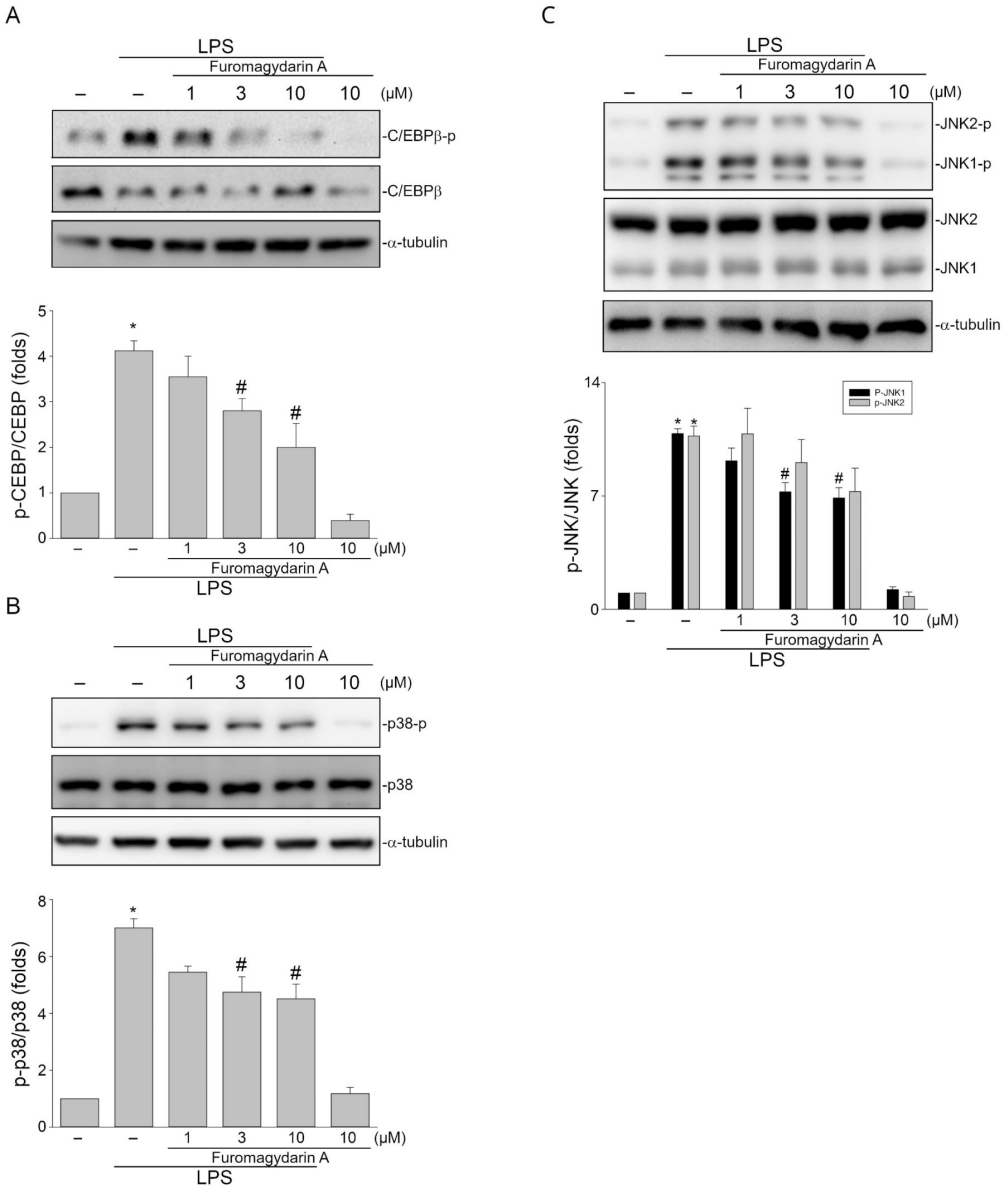
Furomagydarin A reduced LPS-induced C/EBP, p38MAPK or JNK phosphorylation in RAW264.7 macrophages. Cells were treated with furomagydarin A for 30 min, followed by the treatment with LPS (100 ng/ml) for another 30 min. The extent of C/EBP (A), p38MAPK (B) or JNK (C) phosphorylation was determined by immunoblotting. Each column represents the mean ± SEM of six independent experiments. *p < 0.05, compared with the control group; #*p* < 0.05, compared with the group treated with LPS alone.

It appears that furomagydarin A may inhibit LPS-induced activation of transcription factors that lead to COX-2 expression in RAW264.7 macrophages. Many transcription factors including C/EBPβ have been reported to upregulate COX-2 expression in response to a variety of stimuli^9^^,^^26^. Therefore, furomagydarin A was further investigated with its effect on C/EBPβ phosphorylation, believed to be C/EBPβ activation with higher transcriptional activity^22^. As shown in Figure 4(A), LPS caused an increase in C/EBPβ phosphorylation and this effect was significantly reduced by furomagydarin A treatment. In addition, LPS-induced COX-2 expression is also contributed by MAPKs such as p38MAPK and JNK1/2^8^. We noted in this study that furomagydarin A is also capable of suppressing LPS-induced p38MAPK (Figure 4B) and JNK1/2 (Figure 4C) phosphorylation in RAW264.7 macrophages. p38MAPK^26^ or JNK1/2^10^ signalling pathway has been reported contributing to C/EBPβ phosphorylation and activation in response to inflammatory stimuli. It suggests that furomagydarin A suppression of LPS-induced COX-2 expression may attribute to the inhibition of p38MAPK, JNK1/2 and C/EBPβ signalling pathways.

## Conclusions

In summary, from the methanol extract of the seeds of *M. pastinacea* two new benzofuran glucosides (furomagydarin A and B), together with three known coumarins were isolated. In addition, this study highlighted that furomagydarin A was able to reduce the LPS-induced COX-2 expression and to inhibit LPS-induced phosphorylation of p38MAPK, JNK1/2 and C/EBPβ in RAW264.7 macrophages. The underlying mechanisms of furomagydarin A in reducing p38MAPK, JNK1/2 and C/EBPβ phosphorylation remain to be identified. Activation of MKP-1, a protein phosphatase, was recently shown to be causally related to p38MAPK, JNK1/2 or C/EBPβ dephosphorylation and COX-2 expression in cerebral endothelial cells^9^ and RAW264.7 macrophages^8^ after LPS exposure. It raises the possibility that MKP-1 may be responsible for furomagydarin A-induced dephosphorylation of p38MAPK, JNK1/2 or C/EBPβ, as well as COX-2 reduction in LPS-stimulated RAW264.7 macrophages. Taken together, these findings suggest that furomagydarin A may act as a potential agent against inflammatory disorders. The specific mechanisms underlying furomagydarin A-suppressed inflammation and more potent furomagydarin A-derived compounds are worth to be further characterised and developed.

## Supplementary Material

Supplemental Material
