# Supplementary material for: Suppression of lipopolysaccharide-induced COX-2 expression via p38MAPK, JNK, and C/EBPβ phosphorylation inhibition by furomagydarin A, a benzofuran glycoside from Magydaris pastinacea
Source: J Enzyme Inhib Med Chem. 2023 Dec 7;39(1):2287420. doi: 10.1080/14756366.2023.2287420 (PMC11792810; doi:10.1080/14756366.2023.2287420)
Supplement: Supplemental Material [file IENZ_A_2287420_SM6064.pdf]

Supplementary Material

Suppression of Lipopolysaccharide-induced COX-2 Expression via p38MAPK, JNK and C/EBPβ Phosphorylation Inhibition by Furomagydarin A, a Benzofuran Glycoside from Magydaris pastinacea

Shiu-Wen Huang[a,b,c,1], Ming Jen Hsu[a,c,1], Hsiu-Chen Chen[a], Rita Meleddu[d], Simona Distinto[d], Elias Maccioni[d], and Filippo Cottiglia[d]*

[a]Department of Pharmacology, School of Medicine, College of Medicine, Taipei Medical University, Taipei 11031, Taiwan

[b]Department of Medical Research, Taipei Medical University Hospital, Taipei 11031, Taiwan

[c]Graduate Institute of Medical Sciences, College of Medicine, Taipei Medical University, Taipei 11031, Taiwan

[d]Department of Life and Environmental Sciences, University of Cagliari, Cittadella Universitaria di Monserrato, 09042 Monserrato, Italy

*Corresponding author. Tel +39-0706758979; e-mail: cottiglf@unica.it.

1 These authors contributed equally to this work.

*Corresponding author. Tel +39-0706758979; fax +39-0706758551; e-mail: cottiglf@unica.it

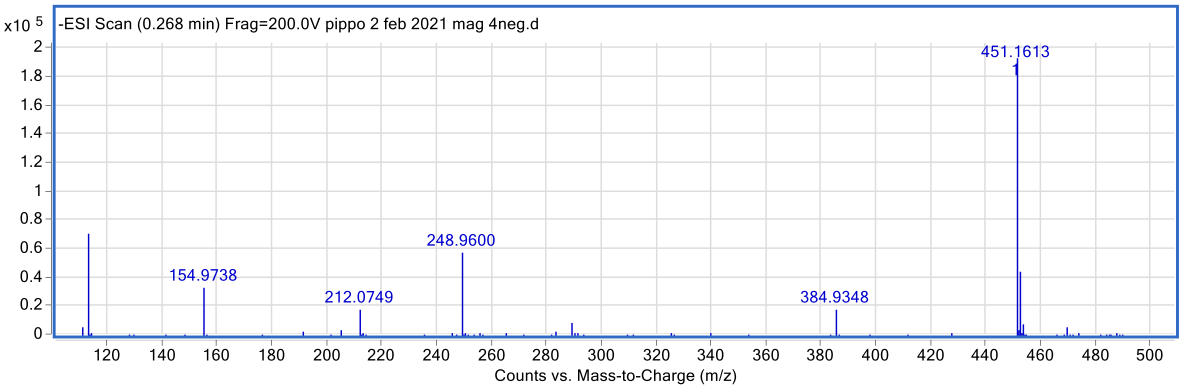

Figure S1: HR ESIMS spectrum (negative mode) of furomagydarin A (**1**)

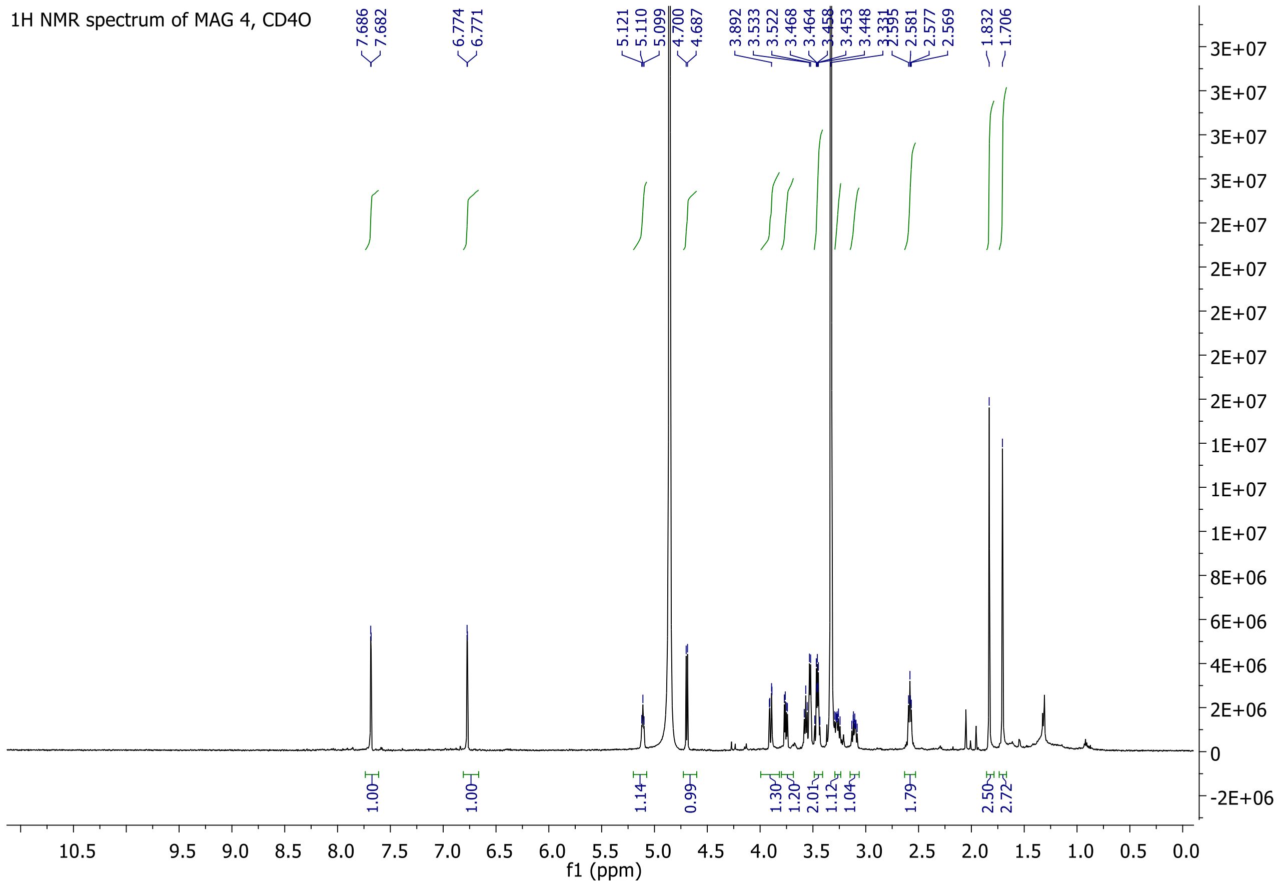

Figure S2: ^{1}H NMR spectrum (600 MHz, CD_4O) of furomagydarin A (**1**)

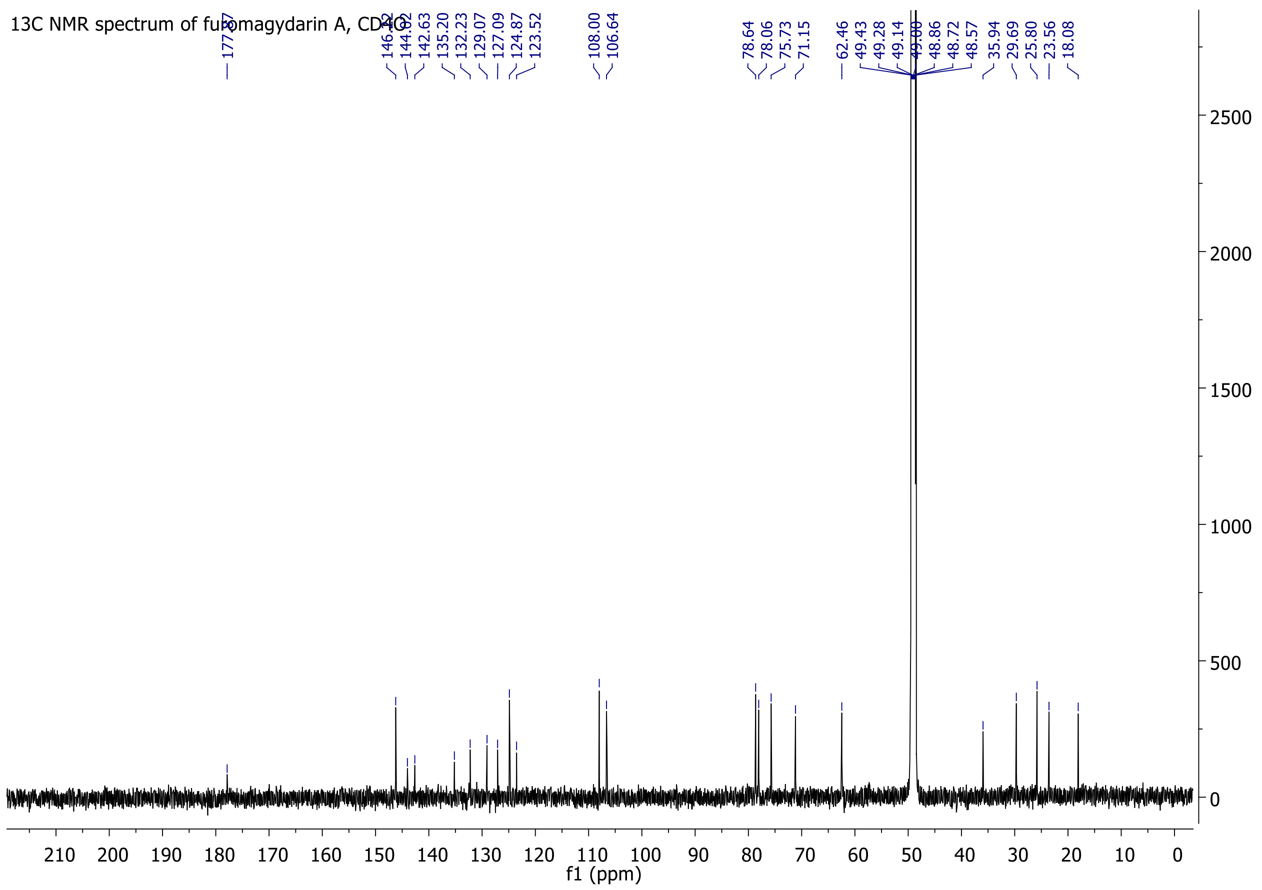

Figure S3: ^{13}C NMR spectrum (100 MHz, CD_4O) of furomagydarin A (**1**)

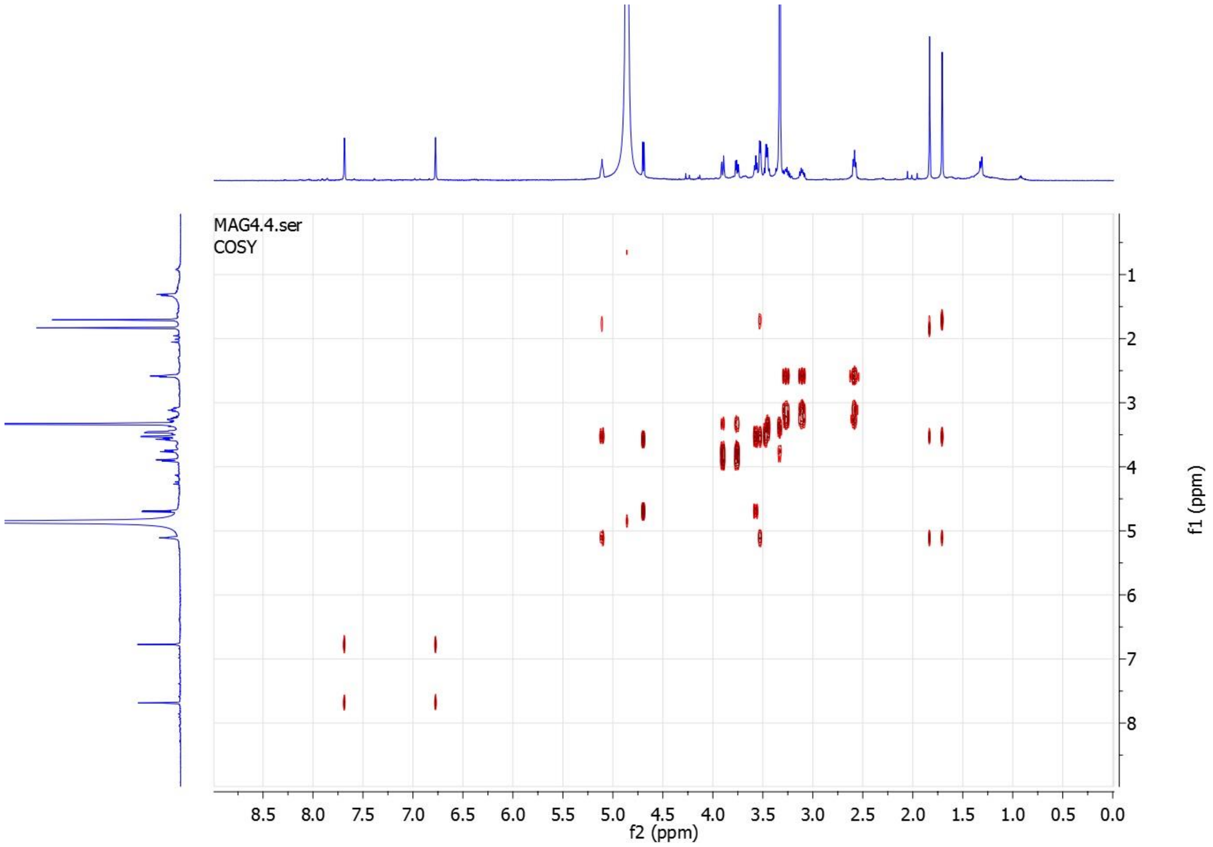

Figure S4: COSY spectrum (600 MHz, CD_4O) of furomagydarin A (**1**)

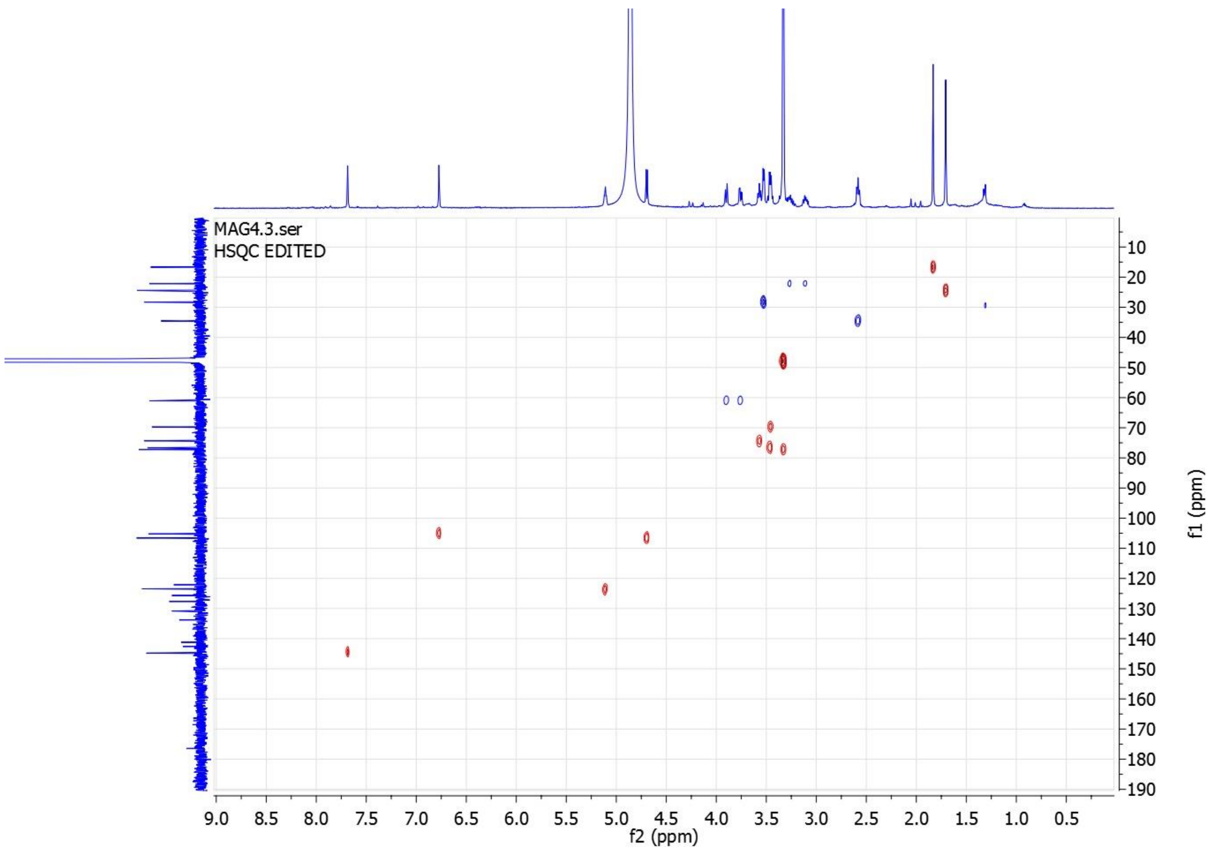

Figure S5: multiplicity-edited HSQC spectrum (600 MHz, CD_4O) of furomagydarin A (**1**)

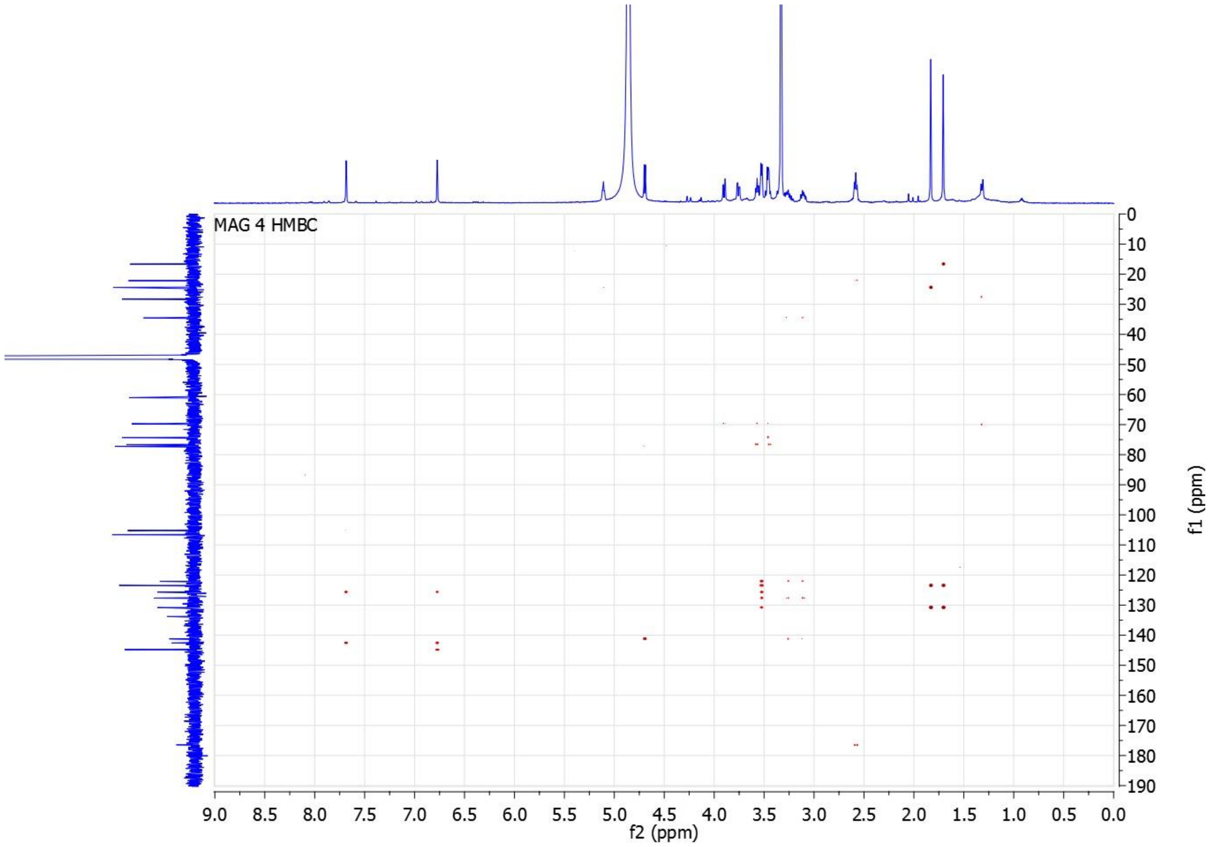

Figure S6: HMBC spectrum (600 MHz, CD_4O) of furomagydarin A (**1**)

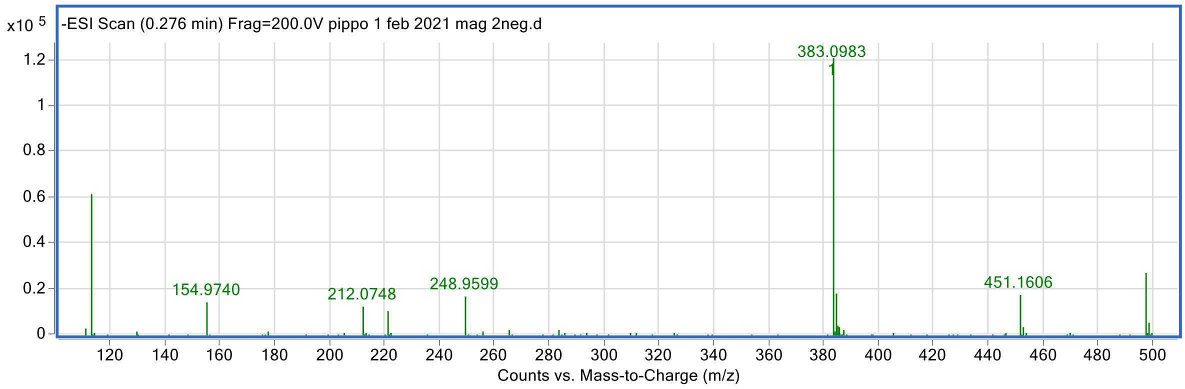

Figure S7: HR ESIMS spectrum (negative mode) of furomagydarin B (**2**)

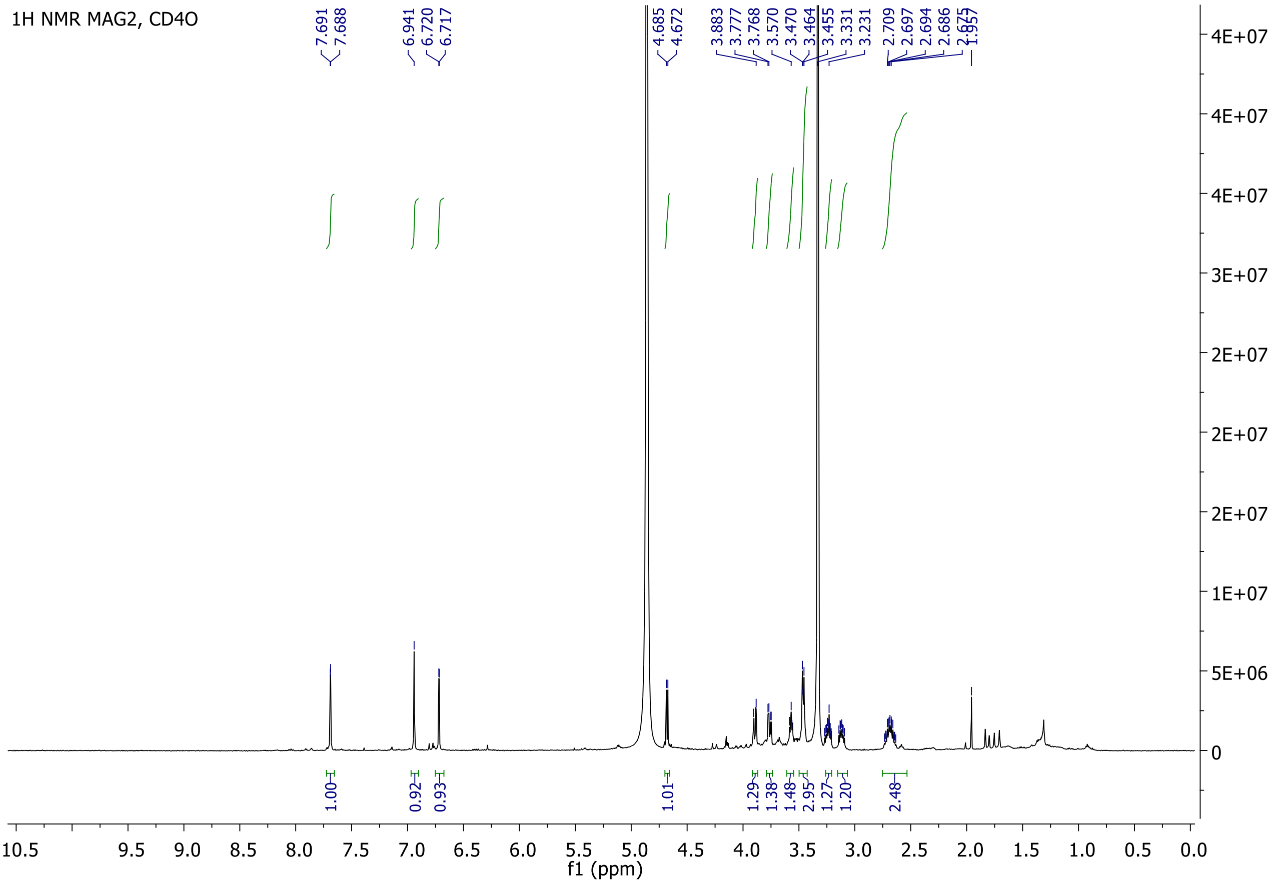

Figure S8: ^{1}H NMR spectrum (600 MHz, CD_4O) of furomagydarin B (**2**)

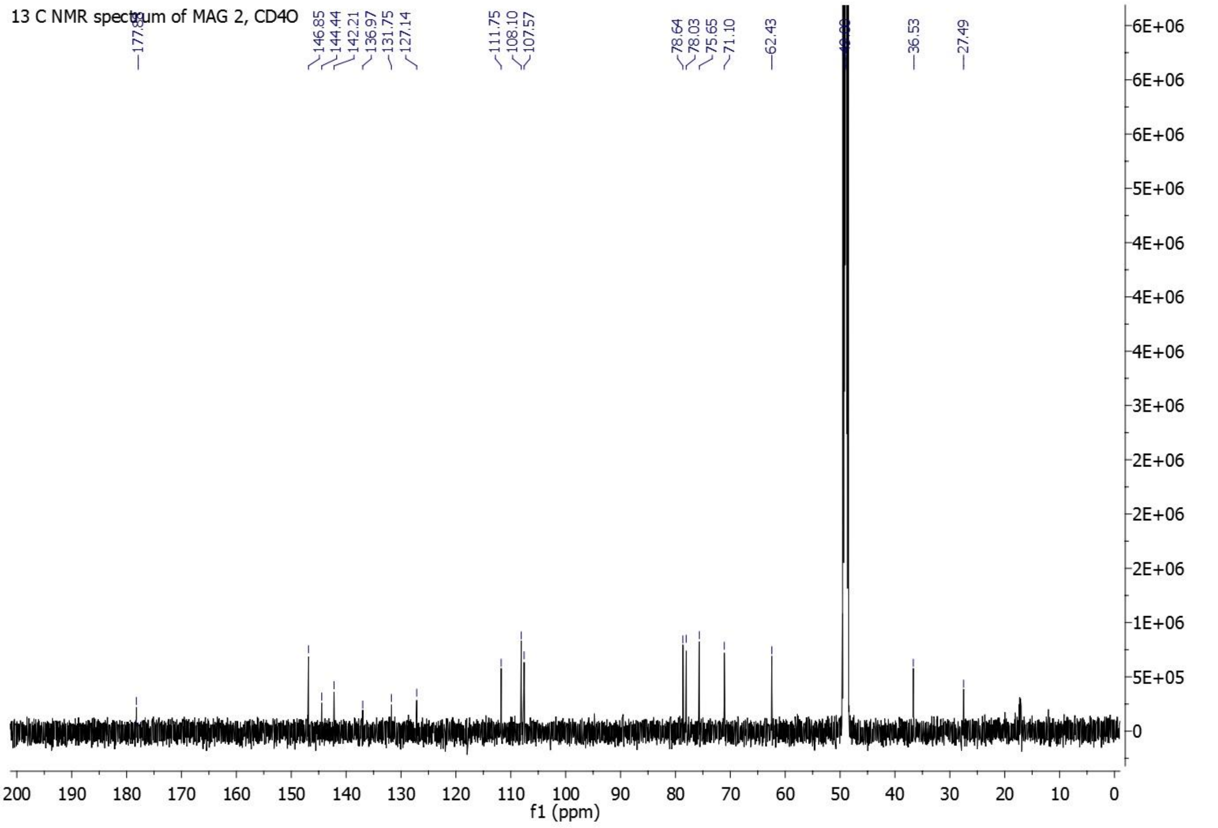

Figure S9: ^{13}C NMR spectrum (600 MHz, CD_4O) of furomagydarin B (**2**)

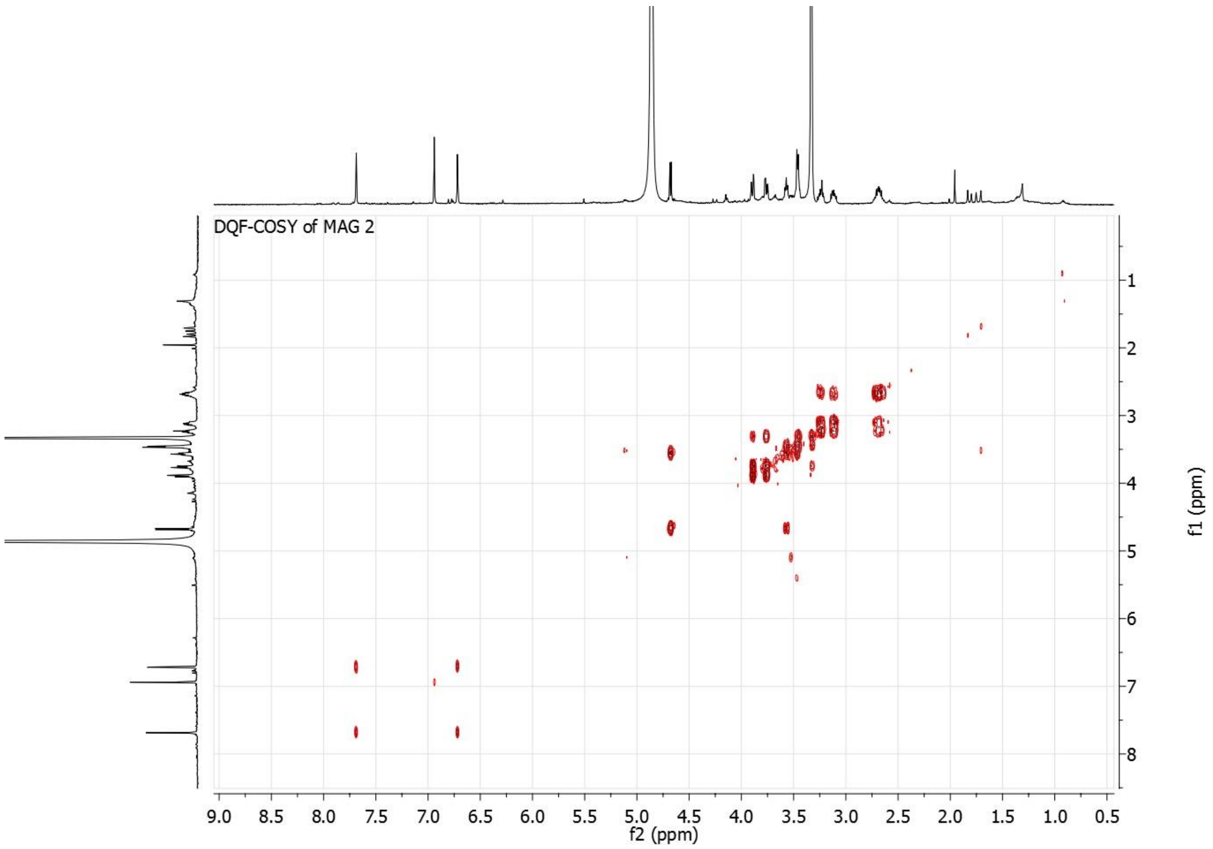

Figure S10: COSY spectrum (600 MHz, CD_4O) of furomagydarin B (**2**)

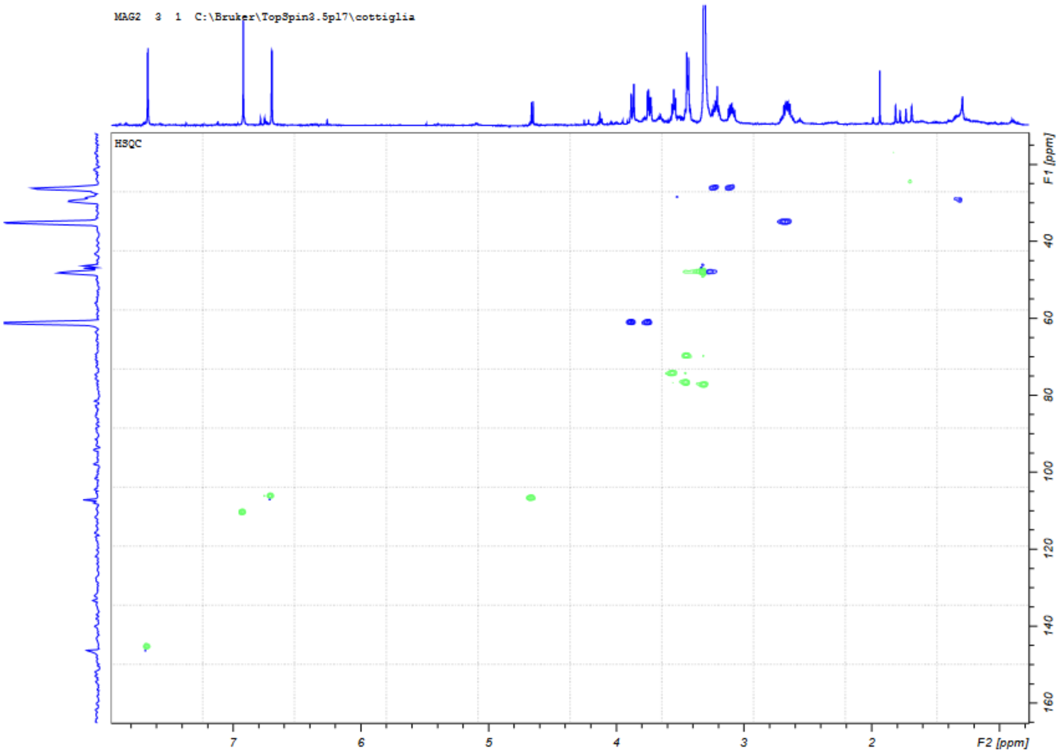

Figure S11: multiplicity-edited HSQC spectrum (600 MHz, CD_4O) of furomagydarin B (**2**)

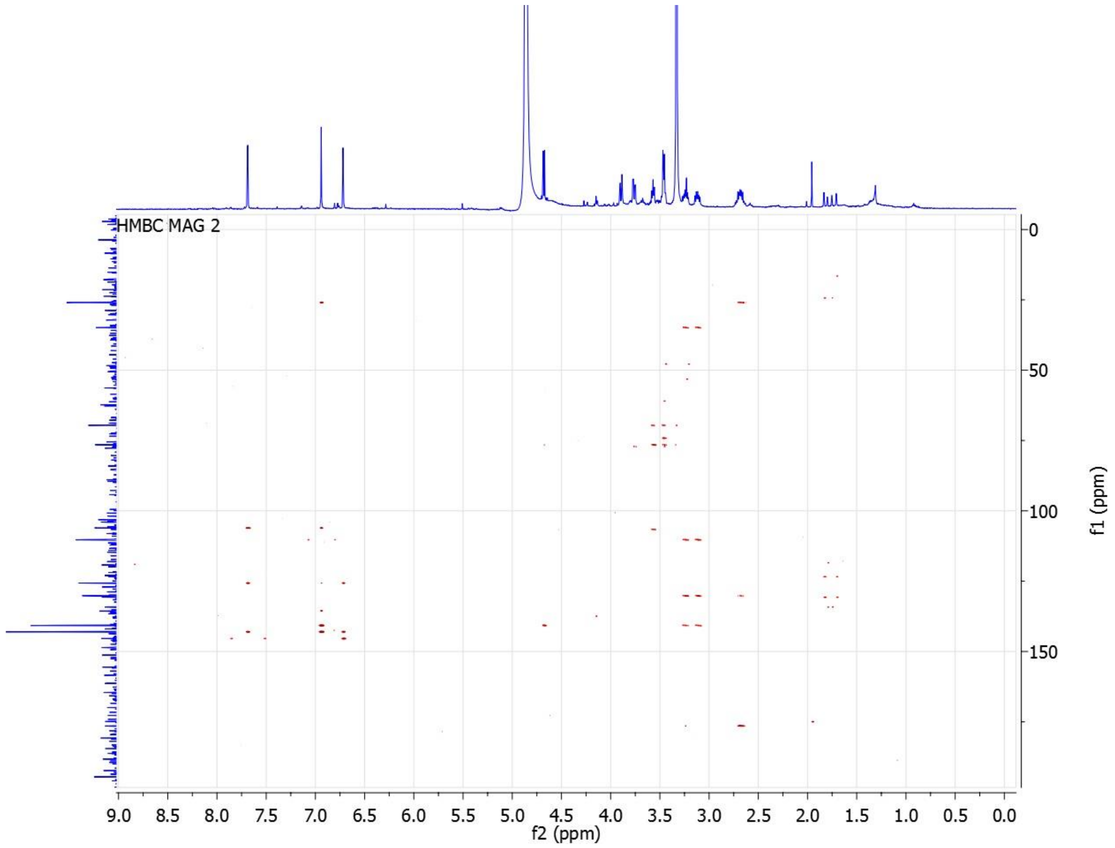

Figure S12: HMBC spectrum (600 MHz, CD_4O) of furomagydarin B (**2**)
